# Comparative Analysis of Alcohol Control Policies in 30 Countries

**DOI:** 10.1371/journal.pmed.0040151

**Published:** 2007-04-24

**Authors:** Donald A Brand, Michaela Saisana, Lisa A Rynn, Fulvia Pennoni, Albert B Lowenfels

**Affiliations:** 1 Department of Medicine, New York Medical College, Valhalla, New York, United States of America; 2 Department of Pediatrics, New York Medical College, Valhalla, New York, United States of America; 3 School of Public Health, New York Medical College, Valhalla, New York, United States of America; 4 Joint Research Centre, European Commission, Ispra, Italy; 5 New York Medical College, Valhalla, New York, United States of America; 6 Department of Statistics, University of Milano-Bicocca, Italy; 7 Department of Surgery, New York Medical College, Valhalla, New York, United States of America; 8 Department of Community and Preventive Medicine, New York Medical College, Valhalla, New York, United States of America; James Cook University, Australia

## Abstract

**Background:**

Alcohol consumption causes an estimated 4% of the global disease burden, prompting goverments to impose regulations to mitigate the adverse effects of alcohol. To assist public health leaders and policymakers, the authors developed a composite indicator—the Alcohol Policy Index—to gauge the strength of a country's alcohol control policies.

**Methods and Findings:**

The Index generates a score based on policies from five regulatory domains—physical availability of alcohol, drinking context, alcohol prices, alcohol advertising, and operation of motor vehicles. The Index was applied to the 30 countries that compose the Organization for Economic Cooperation and Development and regression analysis was used to examine the relationship between policy score and per capita alcohol consumption. Countries attained a median score of 42.4 of a possible 100 points, ranging from 14.5 (Luxembourg) to 67.3 (Norway). The analysis revealed a strong negative correlation between score and consumption (*r* = −0.57; *p* = 0.001): a 10-point increase in the score was associated with a one-liter decrease in absolute alcohol consumption per person per year (95% confidence interval, 0.4–1.5 l). A sensitivity analysis demonstrated the robustness of the Index by showing that countries' scores and ranks remained relatively stable in response to variations in methodological assumptions.

**Conclusions:**

The strength of alcohol control policies, as estimated by the Alcohol Policy Index, varied widely among 30 countries located in Europe, Asia, North America, and Australia. The study revealed a clear inverse relationship between policy strength and alcohol consumption. The Index provides a straightforward tool for facilitating international comparisons. In addition, it can help policymakers review and strengthen existing regulations aimed at minimizing alcohol-related harm and estimate the likely impact of policy changes.

## Abstract 2

Using an index that gauges the strength of national alcohol policies, a clear inverse relationship was found between policy strength and alcohol consumption.

## Editors' Summary

**Background.:**

Alcohol drinking is now recognized as one of the most important risks to human health. Previous research studies (see the research article by Rodgers et al., linked below) have predicted that around 4% of the burden of disease worldwide comes about as a result of drinking alcohol, which can be a factor in a wide range of health problems. These include chronic diseases such as cirrhosis of the liver and certain cancers, as well as poor health resulting from trauma, violence, and accidental injuries. For these reasons, most governments try to control the consumption of alcohol through laws, although very few countries ban alcohol entirely.

**Why Was This Study Done?:**

Although bodies such as the World Health Assembly have recommended that its member countries develop national control policies to prevent excessive alcohol use, there is a huge variation between national policies. It is also very unclear whether there is any link between the strictness of legislation regarding alcohol control in any given country and how much people in that country actually drink.

**What Did the Researchers Do and Find?:**

The researchers carrying out this study had two broad goals. First, they wanted to develop an index (or scoring system) that would allow them and others to rate the strength of any given country's alcohol control policy. Second, they wanted to see whether there is any link between the strength of control policies on this index and the amount of alcohol that is drunk by people on average in each country. In order to develop the alcohol control index, the researchers chose five main areas relating to alcohol control. These five areas related to the availability of alcohol, the “drinking context,” pricing, advertising, and vehicles. Within each policy area, specific policy topics relating to prevention of alcohol consumption and harm were identified. Then, each of 30 countries within the OECD (Organization for Economic Cooperation and Development) were rated on this index using recent data from public reports and databases. The researchers also collected data on alcohol consumption within each country from the World Health Organization and used this to estimate the average amount drunk per person in a year. When the researchers plotted scores on their index against the average amount drunk per person per year, they saw a negative correlation. That is, the stronger the alcohol control policy in any given country, the less people seemed to drink. This worked out at around roughly a 10-point increase on the index equating to a one-liter drop in alcohol consumption per person per year. However, some countries did not seem to fit these predictions very well.

**What Do These Findings Mean?:**

The finding that there is a link between the strength of alcohol control policies and amount of alcohol drinking does not necessarily mean that greater government control causes lower drinking rates. The relationship might just mean that some other variable (e.g., some cultural factor) plays a role in determining the amount that people drink as well as affecting national policies for alcohol control. However, the index developed here is a useful method for researchers and policy makers to measure changes in alcohol controls and therefore understand more clearly the factors that affect drinking rates. This study looked only at the connection between control measures and extent of alcohol consumption, and did not examine alcohol-related harm. Future research might focus on the links between controls and the harms caused by alcohol.

**Additional Information.:**

Please access these Web sites via the online version of this summary at http://dx.doi.org/10.1371/journal.pmed.0040151.

- A Perspective in *PLoS Medicine* by Alison Ritter accompanies this article: “Comparing alcohol policies between countries: Science or silliness?”
- Facts and figures on alcohol are available from the World Health Organization, including information about the burden of disease worldwide as a result of alcohol
- Information from the US Centers for Disease Control and Prevention is available about alcohol and public health
- A 2004 *PLoS Medicine* research article includes discussion of the health burdens of alcohol: Rodgers A, Ezzati M, Vander Hoorn S, Lopez AD, Lin RB, et al. (2004) Distribution of major health risks: Findings from the global burden of disease study. *PLoS Medicine* 1(1): e27. doi:10.1371/journal.pmed.0010027
- Current information about research on alcohol and alcoholism is available from the National Institute on Alcohol Abuse and Alcoholism

## Introduction

Alcohol consumption contributes to more than 60 health problems that cause an estimated 4% of the global disease burden [1,2]. International differences in the occurrence of alcohol-associated disease derive from a complex interaction of drinking patterns, total alcohol consumption, and societal priorities. Governments impose various regulations to mitigate the adverse effects of alcohol while attempting to respect individuals' rights to consume alcohol in moderation [3].

The history of alcohol control policy dates back more than 3,000 years. After World War I, many countries initiated and soon repealed laws prohibiting the sale of alcoholic beverages. Modern efforts to prevent alcohol problems through public policy received wide recognition with publication of a 1975 monograph, *Alcohol Control Polices in Public Health Perspective,* sponsored by the World Health Organization [4]. This report led to a World Health Assembly recommendation that countries design national alcohol polices emphasizing preventive measures [5].

**Table 1:**
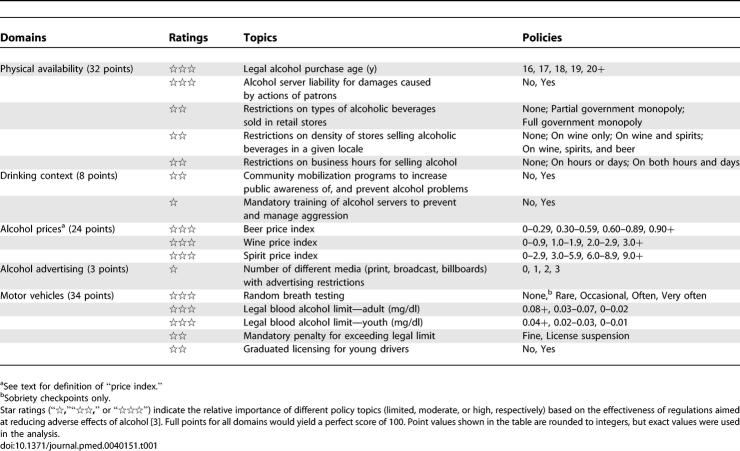
Components of the Alcohol Policy Index

To assist public health leaders and policymakers, we developed a composite index that measures the strength of a country's alcohol control policies, applied the index to the 30 countries included in the Organization for Economic Cooperation and Development, evaluated the robustness of the index, and examined the relationship between index value and per capita alcohol consumption.

## Methods

### Policy Topics

We examined five regulatory domains identified in an analysis of alcohol research and public policy sponsored by the World Health Organization: physical availability of alcohol, drinking context, alcohol prices, alcohol advertising, and operation of motor vehicles (Table 1) [3]. We focused on 16 policy topics within these domains because alcohol control policies related to these topics have shown potential for reducing adverse effects of alcohol. We excluded topics for which even strict policies have not been proven effective (e.g., warning labels on alcoholic beverage containers). We also excluded topics pertaining to the treatment of problem drinkers because our investigation focused on public health measures aimed at prevention. Finally, we excluded interventions that are not currently used in any of the 30 countries (e.g., total prohibition) and topics related to the enforcement of existing policies (reliable data on enforcement are not available for many of these countries).

**Table 2:**
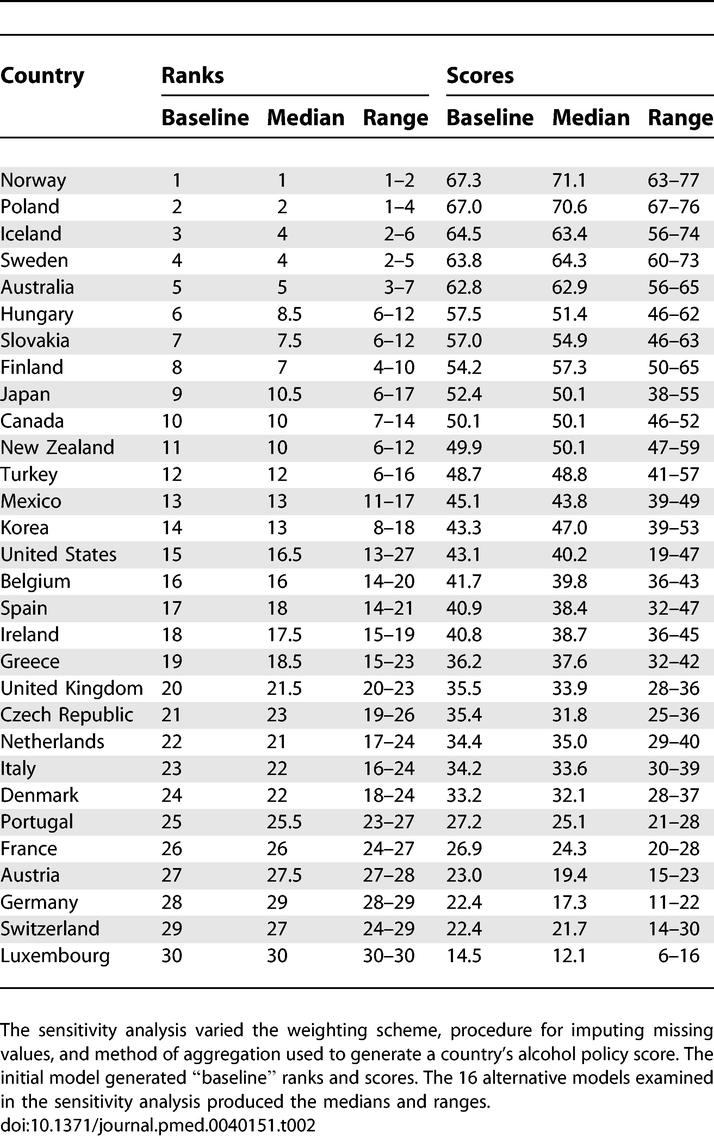
Results of Sensitivity Analysis

### Data Sources

We chose the Organization for Economic Cooperation and Development because its 30 member countries are geographically widespread and their alcohol policies are reasonably accessible. We obtained the most current public policy data available from published reports [6–8] and publicly available databases maintained by the World Health Organization [9,10] and by individual countries (complete list of references available from the authors). All data were published between 2000 and 2005. If the above sources failed to provide information about a given policy, we contacted public health officials or other knowledgeable authorities from the country in question. We obtained alcohol consumption rates for each country from the World Health Organization [10] and a standard reference book on alcohol consumption [11]. Those sources report total liters of ethanol consumed in each country divided by the most recent estimate of the country's mid-year population. The sources estimate consumption of beer, wine, and spirits from sales data supplied by industry or tax receipts, using a conversion coefficient to approximate the amount of pure alcohol contained in each beverage: 0.045 for beer, 0.129 for wine, and 0.411 for spirits. We used the most recent consumption data available: 2003 data for 28 countries and 2001 data for the remaining 2 countries (Korea and Turkey).

In the United States, alcohol regulations vary from state to state. We therefore obtained policy data from three widely separated, ethnically diverse states (New York, Michigan, and California) [12] and combined data from these states to represent U.S. policy. These three states, which rank among the ten most populous states, have a combined population of 65 million, or 22% of the total U.S. population. To avoid introducing any deliberate bias, we selected these states before examining their alcohol policies or consumption data. Together, we believe that they adequately represent the average situation in US, but we recognize that other states might have produced somewhat different results.

### Alcohol Policy Index

We developed an “Alcohol Policy Index” that generated scores with a potential range of 0 to 100 points, and used the Index to assign a score to each country. The scoring system weighted different topics according to the effectiveness of regulations aimed at reducing adverse effects of alcohol as reported in the World Health Organization's recent alcohol policy study [3]. Based on the available scientific evidence, that report assigned a “star” rating to each topic, using one, two, or three stars (“⋆,” “⋆⋆,” or “⋆⋆⋆”) to indicate limited, moderate, or high effectiveness (Table 1). We assigned a weight of 1, 2, or 3 to each topic according to its star rating, then determined that the proportionate point values 2.6, 5.3, and 7.9 would yield a total of 100 points when summed over the 16 topics (2.6 points × 2 topics + 5.3 points × 6 topics + 7.9 points × 8 topics = 100 points). These point values have been rounded to the nearest decimal point in this report, but exact values were used in the analysis. For a given topic, a country received credit based on the strictness of the country's own policy relative to that topic: no points for the most lenient policy option, full points for the most restrictive option, and partial points for intermediate options. For example, legal alcohol purchase ages of 16, 17, 18, 19, or 20+ y generate 0, 2.0, 3.9, 5.9, or 7.9 points, respectively, corresponding to 0%, 25%, 50%, 75%, or 100% credit for this three-star topic. Summing the points credited to a country over all topics in a given domain yields the domain score; summing the domain scores yields the country's overall alcohol policy score.

In Table 1, the “price index” for an alcoholic beverage refers to the retail price (including alcohol taxes) for a standard size beverage container (0.5-l beer, 0.75-l wine, or 0.75-l spirits) adjusted for a country's standard of living. The adjustment consists of dividing the retail price by the per capita share of a country's gross domestic product (GDP), and multiplying the result by 10,000 to produce a price index with an approximate range of 0 to 20 [13]. That is,

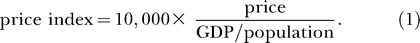



### Statistical Methods

Of the 480 policies of interest (16 topics × 30 countries), we were able to ascertain information about 453 policies (94.4%). In the analysis, we handled missing policy data by substituting the mean point value credited to countries with *known* policies for the topic in question [14].

By acknowledging various methodological assumptions that are intrinsic to policy research, a sensitivity analysis can determine if results change substantially when those assumptions are varied over a reasonable range of possibilities [15,16]. To evaluate the robustness of the Alcohol Policy Index, we varied assumptions involving the weighting scheme, the imputation of missing values, and the method of aggregating data from different policy topics. In the sensitivity analysis, we refer to the initial set of assumptions as the “baseline model.”

#### Weighting.

We tested four different weighting schemes: baseline weighting (weights 1, 2, and 3 applied to one-star, two-star, and three-star topics, respectively), heavy weighting (weights 1, 3, and 5 used instead of 1, 2, and 3), equal weighting (same weight for all topics), and “country-specific weighting.” The last alternative, also known as data envelopment analysis, involved choosing a set of weights for each country in a manner that maximized that country's performance relative to all other countries [17]. This best-case scenario was included to discourage countries from rejecting the Alcohol Policy Index on grounds that a given weighting scheme might not be fair to a particular country. In applying a country-specific method, it is essential to place reasonable bounds on the weights; otherwise, a country could achieve a perfect score simply by assigning zero weight to all topics for which the government had not implemented the strictest policy option. To preclude this possibility, we required minimum and maximum weights to differ by no more than a factor of 12—that is, four times the spread of the weights used in the baseline model.

#### Imputation.

The baseline model uses mean-value substitution to impute missing policy data (5.6% of all items). In the sensitivity analysis, we used a more refined approach, known as “nearest-neighbor” imputation. This method computes the mathematical “distance” between every pair of countries based on all shared (nonmissing) policy data. Each missing item is then replaced by the value of the corresponding item from the country's nearest neighbor; that is, the country that is mathematically closest (most similar with respect to its policies) to the one with the missing item. If a country has more than one nearest neighbor, the mode (most frequent value) from those neighbors is used as the replacement [14].

#### Aggregation.

The Alcohol Policy Index generates a score by adding together weighted contributions from each of 16 policy topics to permit the ranking of countries based on their aggregate scores. Some policy analysts have challenged aggregations based on additive models because of inherent theoretical inconsistencies [18,19]. In the sensitivity analysis, we applied an alternate approach, a “noncompensatory” method that overcomes some of the inconsistencies of additive models [20,21]. This alternative approach examines every pair of countries to determine which country performs better on each topic, ignoring the size of the difference in strictness of their policies. When two countries have equivalent policies, the method splits the credit for that topic equally between the two countries. Under this method, a country cannot fully “compensate” for a preponderance of weak policies with a small number of exceptionally strong policies. In other words, to attain a reasonably good score under a noncompensatory method, a country must devote a reasonable amount of attention to all policy topics. This is not true under additive models, which are fully compensatory.

For the present analysis, we adapted a previously published noncompensatory ranking algorithm [21], modifying the algorithm to generate not only a ranking but also a set of scores that could be compared with the baseline Alcohol Policy Index scores. Specifically, our noncompensatory algorithm computed scores as follows.

Let *n_ij_* ≡ number of countries compared with which country *i* has a stricter policy relative to topic *j*, and let *k_ij_* ≡ number of countries compared with which country *i* has an equivalent policy relative to topic *j*, where 0 ≤ *n_ij_* ≤ 29, 0 ≤ *k_ij_* ≤ 29, 1 ≤ *i* ≤ 30, and 1 ≤ *j* ≤ 16; *w_j_* ≡ weight assigned to topic *j*; *S_i_* ≡ the “noncompensatory score” for country *i*, where

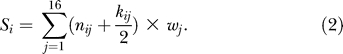
To implement the sensitivity analysis, we recalculated scores using each of the 16 combinations of assumptions (four weighting schemes × two methods of imputation × two methods of aggregation). Each combination—or “scenario”—produced its own ranking. To facilitate comparisons, we calibrated the scores derived from each scenario to produce equivalent ranges. For each country, we then identified the median rank and score of all 16 scenarios and compared those medians with the corresponding baseline values using the Pearson or Spearman correlation coefficient, as appropriate. We also computed the correlations using extreme values in place of medians. These coefficients provided a measure of the robustness of the Alcohol Policy Index.

To investigate a possible relationship between policy score and per capita alcohol consumption, we produced a scatter plot of score versus consumption and performed a simple linear regression of the two variables, using the Pearson correlation coefficient to test for a nonzero slope.

## Results

**Figure 1:**
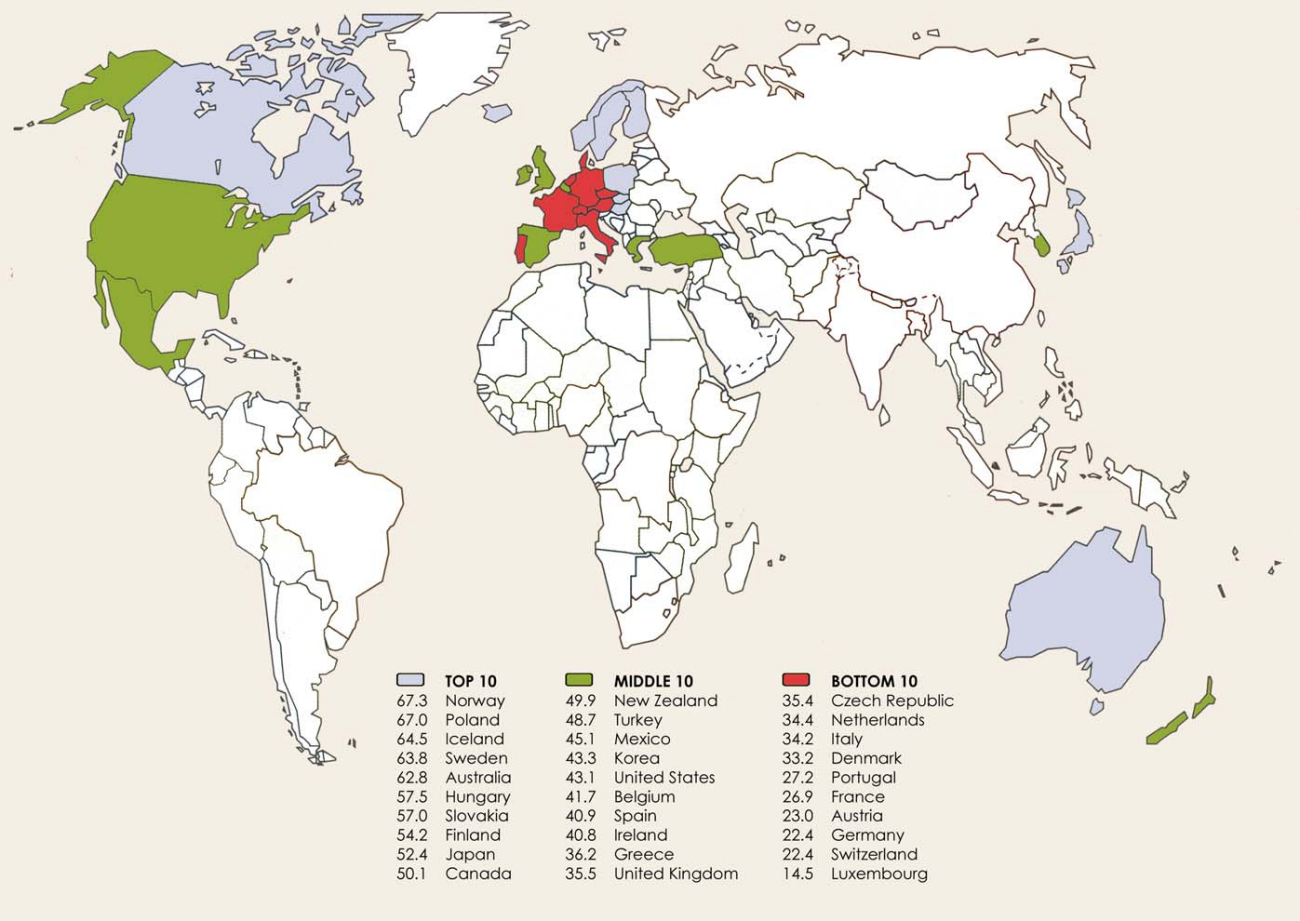
Alcohol Policy Scores of the 30 Countries Included in the Organization for Economic Cooperation and Development

Countries attained a median score of 42.4, ranging from 14.5 (Luxembourg) to 67.3 (Norway; Figure 1). In spite of rather extreme methodological assumptions considered in the sensitivity analysis, relatively few countries (seven of 30) shifted more than five positions under any scenario (Table 2). It follows that most countries were not markedly affected by the choice of assumptions used to calculate scores. Median ranks and scores from the 16 scenarios varied hardly at all from baseline values (*r* = 0.99 for ranks as well as scores). For 29 of the 30 countries, median and baseline ranks differed by no more than two positions. For one country—Hungary—they differed by 2.5 positions. Even when baseline ranks and scores were compared with the extremes from the 16 scenarios—that is, with the ranks and scores that deviated most from baseline—the correlation coefficients were 0.87 for ranks and 0.92 for scores (*p* < 0.001 for each). These results suggest that the baseline Alcohol Policy Index is a reliable summary measure (for both ranks and scores) that is not biased against particular methodological scenarios.

**Figure 2:**
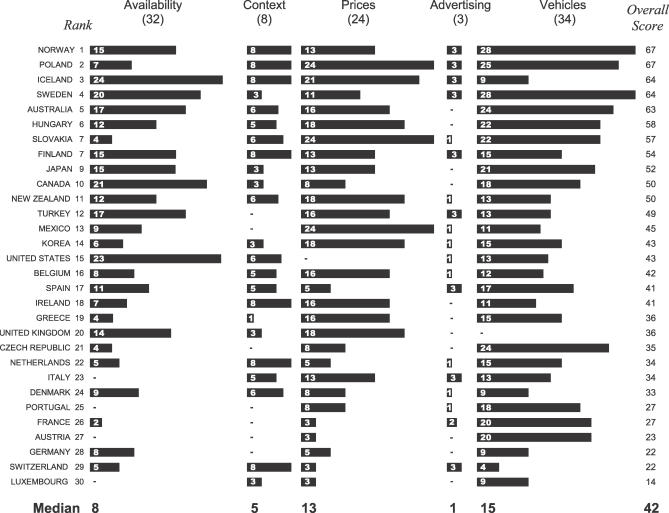
Point Breakdown of Alcohol Policy Scores by Regulatory Domain Bar lengths indicate points credited to countries for alcohol control policies in each of five regulatory domains (physical availability of alcohol, drinking context, alcohol prices, alcohol advertising, and motor vehicles). –, zero points in a given domain. Points do not always add up to overall scores due to rounding errors. Numbers in parentheses indicate the full point value of each domain. Median scores within each domain and the overall median appear beneath the bars.

Figure 2 summarizes each country's performance by regulatory domain. Countries received a median domain score of 8 out of a possible 32 points (i.e., 25% credit) for policies involving physical availability, 5 of 8 points (63%) for the drinking context, 13 of 24 points (54%) for alcohol prices, 1 of 3 points (33%) for alcohol advertising, and 15 of 34 points (44%) for policies pertaining to motor vehicles. It follows that there is considerable room for improvement in all regulatory domains.

**Figure 3:**
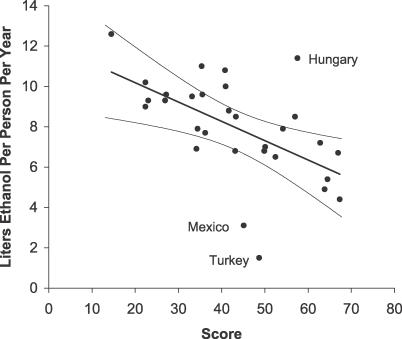
Score versus Alcohol Consumption Scatter plot shows the relationship between alcohol policy score and annual per capita alcohol consumption. The regression line has a slope of −0.10 (*p* = 0.001), signifying a decrease in consumption of 1 l absolute alcohol for each 10-point increase in the score (95% CI 0.4–1.5 l). Arcs show 95% confidence limits. See Discussion for comments about the three identified countries.

The plot of score versus annual per capita alcohol consumption revealed a strong inverse relationship (Figure 3). The Pearson correlation coefficient was −0.57 (*p* = 0.001), and the slope of the regression line was −0.10 (95% confidence interval [CI], −0.15 to −0.04), signifying a decrease in consumption of 1.0 l of alcohol per person per year for each 10-point increase in the score. To factor out the price–demand effect, we recalculated the scores after excluding alcohol prices from the model and then recomputed the correlation. Excluding prices had only a modest effect: the revised correlation coefficient was −0.49 (*p* = 0.006), and the slope was −0.08 (95% CI, −0.14 to −0.03). In the sensitivity analysis, the negative correlation between score and consumption remained strong under all 16 scenarios, ranging from −0.66 to −0.51 (*p* < 0.001 in every case).

## Discussion

The World Health Organization places a high priority on controlling alcohol-related problems through effective economic and public health measures. Nevertheless, our Alcohol Policy Index revealed wide variation in the strength of alcohol control policies among the 30 countries in Europe, Asia, North America, and Australia that constitute the Organization for Economic Cooperation and Development. These countries received scores ranging from 14 to 67 out of a possible 100 points.

### Validity of the Alcohol Policy Index

We subjected the Alcohol Policy Index to two types of validity testing. First, we conducted a sensitivity analysis to determine the effect of varying several methodological assumptions on the scores and ranks generated by the Index. The effect proved to be minimal: median ranks and scores produced by 16 different combinations of assumptions varied little from baseline ranks and scores. It follows that the baseline Alcohol Policy Index gives a fair representation of all scenarios.

Second, we examined the relationship between score and per capita alcohol consumption. We found a strong negative correlation that implied a decrease in consumption of one liter of absolute alcohol per year for each 10-point increase in the score. A few apparent outliers—Mexico, Turkey, and Hungary—deserve comment (Figure 3). Mexico's relatively low level of alcohol consumption may be explained by a high estimated amount of unrecorded consumption (up to 50% of the total) [22]. The discrepancy between Turkey's very low alcohol consumption and its mid-range score may reflect religious opposition to alcohol among the country's predominantly Islamic population. We are not certain why Hungary has high consumption relative to its score, but findings of one policy analysis [23] suggest that Hungary may be an example of a country where relatively strong laws are poorly enforced.

### Implications

The Alcohol Policy Index provides a means for governments and public health leaders to estimate the potential impact of policy changes. If, for example, the United States imposed taxes that raised alcohol prices by 50%, its alcohol policy score would increase from 43 to 48, a change that would theoretically cause a 7% drop in alcohol consumption (95% CI, 3%–10% drop) based on the slope of the regression line in Figure 3. After such a price increase, the U.S. price index would still fall below 22 of the 29 remaining countries. If an individual country could match the best performance of any of the 30 countries in all five domains, that country would attain a score of 86 and achieve a theoretical annual per capita alcohol consumption of 3.9 l (95% prediction interval, 1.8–5.9 l).

### Previous Research

Two recent investigations used composite scoring systems based on expert opinion to evaluate existing alcohol control policies: one published study comparing 25 countries in the Americas [24], and one report comparing 30 European countries [25]. The authors concluded that most governments could reduce alcohol-related problems by strengthening alcohol policy, and that such changes are likely to be cost-effective. These studies did not include a sensitivity analysis or compare alcohol policy with consumption.

### Study Limitations

Although the 30 countries we studied represent four continents, they comprise only 16% of the world's countries. As a result, one must be cautious about generalizing the reported findings. In some regions, for example, unrecorded alcohol consumption represents a substantial fraction of the true total (e.g., certain African nations) [13]. When applying the Index in various parts of the world, therefore, policy analysts will need to recognize that unrecorded amounts may alter the observed relationship between score and consumption, since most of that alcohol would elude regulatory restrictions. Further research is needed to test the model more widely.

The Alcohol Policy Index does not consider the extent to which different countries enforce their existing regulations. Strict policies that are poorly enforced may be less effective than weaker policies that are well enforced. For example, although the 1984 National Minimum Drinking Age Act established 21 years as the minimum in the United States, survey data indicate that, in a given month, 43% of high school students consume alcohol and 10% drive a car after drinking [26]. Absence of enforcement data represents a limitation of the present analysis. Also, the correlation analysis does not consider the exact date of enactment of individual policies in each country. For some policies there may be a lag between enactment and impact on consumption and alcohol-related harm.

Culture is an important determinant of the level and pattern of alcohol consumption. It can affect the proportion of heavy drinkers in a country and may have a significant impact on alcohol-related harm [27]. Such cultural effects would not be captured in an index that focuses on government regulations.

Since this is a cross-sectional study, one cannot infer a causal relationship between policy score and alcohol consumption based on the observed correlation. Nevertheless, longitudinal data suggest that strong regulations reduce consumption. For example, following implementation of Mikhail Gorbachev's strict alcohol policy in 1985, consumption in the Soviet Union dropped and life expectancy increased [28]. Subsequent relaxation of this policy was followed by a sharp increase in consumption and in alcohol-related mortality [29].

A final limitation of our study concerns the use of alcohol consumption rather than alcoholism or alcohol-related harm as the dependent variable in the correlation analysis. While many alcohol control policies aim to limit consumption, consumption per se is not the ultimate concern. Rather, societies want to limit harm associated with excessive or inappropriate alcohol use, including alcoholism. Future studies should therefore examine the relationship between the score generated by the Index and alcohol-related morbidity and mortality.

In summary, this study documented wide variation in the strength of the alcohol-control policies of 30 countries located in Europe, Asia, North America, and Australia. The Alcohol Policy Index, a simple model corroborated using advanced statistical techniques, provides a straightforward tool for facilitating international comparisons. In addition, it can help policymakers review and strengthen existing regulations aimed at minimizing alcohol-related harm and estimate the likely impact of policy changes.

## Abbreviations

CI: confidence interval
